# Social interaction-induced fear memory reduction: exploring the influence of dopamine and oxytocin receptors on memory updating

**DOI:** 10.1038/s41398-024-02955-3

**Published:** 2024-06-06

**Authors:** Angel David Arellano Perez, Aline Sartori Kautzmann, Lucas de Oliveira Alvares

**Affiliations:** 1 Departamento de Biofísica, Laboratório de Neurobiologia da Memória, Instituto de Biociências, Universidade Federal do Rio Grande do Sul, Porto Alegre, Brazil; 2 Programa da Pós-Graduação em Neurociências. Instituto de Ciências Básicas da Saúde (ICBS). Universidade Federal do Rio Grande do Sul, Porto Alegre, Brazil

**Keywords:** Hippocampus, Psychology

## Abstract

It has been well established that a consolidated memory can be updated during the plastic state induced by reactivation. This updating process opens the possibility to modify maladaptive memory. In the present study, we evaluated whether fear memory could be updated to less-aversive level by incorporating hedonic information during reactivation. Thus, male rats were fear conditioned and, during retrieval, a female was presented as a social rewarding stimulus. We found that memory reactivation with a female (but not a male) reduces fear expression within-session and in the test, without presenting reinstatement or spontaneous recovery. Interestingly, this intervention impaired extinction. Finally, we demonstrated that this emotional remodeling to eliminate fear expression requires the activation of dopamine and oxytocin receptors during retrieval. Hence, these results shed new lights on the memory updating process and suggests that the exposure to natural rewarding information such as a female during retrieval reduces a previously consolidated fear memory.

## Introduction

Memories play a crucial role in our ability to adapt to the environment, yet those associated with negative emotional experiences have the potential to transform into maladaptive memories. Such memories can significantly compromise the affected person’s quality of life, giving rise to the manifestation of disorders like phobias and post-traumatic stress disorder (PTSD) [1]. It has become clear that a previously consolidated memory can be modified during the induced plastic state after reactivation [2]. Depending on the conditions during retrieval, such as the presence of novelty or prediction error, the fear memory trace might undergo a destabilization process. This renders it more susceptible to alterations in both content and emotional intensity. Consequently, this represents a significant opportunity to attenuate aversive memories through reconsolidation mechanisms [3–5]. Conversely, fear memory extinction involves prolonged exposure to the trauma reminder within a secure environment, leading to the formation of a new memory that inhibits the expression of the original fear memory [6]. However, extinction has limitations, so that fear memory may eventually reemerges with the passage of time (spontaneous recovery), or by the exposure to the aversive stimulus (reinstatement) [7, 8].

Alternatively, reconsolidation allows new information to be added to the original memory trace ([9]; Bonin & De Koninck, 2015; [2, 10, 11, 12]), providing long-lasting reduction in fear expression [13]. Nevertheless, the majority of reconsolidation-based treatments rely on drugs that frequently pose toxicity concerns and are impractical for use in humans. This presents a considerable challenge in translating such treatments into clinical practice Lee et al,. 2006; [13, 14]. Alternatively, positive valence stimuli, such as chocolate, fruit cereals, caffeine, or methylphenidate, administered during reactivation have been shown to update a context previously associated with a foot shock, making this context less aversive, without presenting spontaneous recovery or reinstatement [15–17].

Studies have demonstrated that exposure to a conspecific or partner of a different sex constitutes a rewarding experience. This exposure is linked to an elevation in dopamine levels in the nucleus accumbens during male-female interactions, encompassing approach, investigation, and consummatory behaviors (Canseco-Alba et al., 2022, Dai et al., 2022) [18, 19]. Moreover, oxytocin plays a pivotal role in mediating the rewarding effects of these social interactions [20–22]. Previous studies have demonstrated that presenting a familiar conspecific is more effective than presenting an unfamiliar one for social buffering and reducing freezing expression in male rats [23].

Our hypothesis posits that introducing a female rat during a fear memory reactivation session would be perceived as a socially and sexually rewarding stimulus, facilitating the assimilation of positive information. This integration occurs following a brief memory reactivation, leading to the updating of the aversive memory to a less distressing level through the incorporation of positive elements. As a consequence, this process would reduce the expression of fear responses in males.

## Materials and methods

### Animals

A total of 204 male and female Wistar rats (2–3 months old, weighing approximately 300 g) from CREAL at the Federal University of Rio Grande do Sul (UFRGS) were used for all the experiments. The sample sizes were determined based on pilot experiments, previous research conducted by our research group, and references to related studies in the literature [24]. Animals were housed in Plexiglas boxes (47 × 30 x 20), with 4 animals per cage. To ensure randomization, each rat per cage was assigned to a each group condition. They were maintained on a 12:12 light/dark cycle (7 am/7 pm) at a controlled temperature of 21 °C ± 2, with food and water available ad libitum. Experiments were conducted during the light cycle, between 9 am and 5 pm. All procedures adhered to the Brazilian ethical guidelines for animal research.

### Drugs

SCH23390 (SCH) (Sigma-Aldrich) is a selective antagonist of the D1/5 dopamine receptors. SCH was dissolved in 15% DMSO to obtain a final concentration of 1.25 µg/µL. We used a volume of 1 µl (0.5 µl/side) to be infused 20 min before the reactivation session.

Atosiban (ATO) (Sigma-Aldrich) is a selective antagonist of oxytocin (OXT) receptors. ATO was diluted in 0.9% saline to obtain a final concentration of 1 µg/ul. We administered a volume of 1 µL (0.5 µl/side) 20 minutes before the activation session.

OXT (syntocinon, Mylan Lab) was dissolved in 0.9% saline to obtain a final concentration of 0.033 µg/µL. We infused 1 µl (0.5 µl/side) 25 min before the reactivation session.

All drugs or their vehicle were administered via a pump attached to Hamilton syringes. By bilateral intrahippocampal infusion at a rate of 0.5 μL/min and waited an additional 30 s before removing the infusion needle.

### Social interaction

#### Familiar exposure (30 min)

The social interaction protocol was performed 24 h after the conditioning session, in a different room than the conditioning room. This protocol was developed for rats to create familiarity with another (female or male) non-trained conspecific. It consisted of randomly placing a conditioned rat with an unfamiliar conspecific of the same age (same or different sex) in a Plexiglas box (the same model as the home cage) for 30 min. During the exposure, a low-intensity light of 25 lux was equally distributed in the experimental room. Behavior was recorded by video monitoring. The social interaction protocol was performed in the light cycle phase (Ramirez, et al., 2015) [25]. The animals returned to their cages after the social interaction session.

#### Familiar exposure (overnight)

This group followed the same social interaction protocol mentioned above, but animals remained in the cage with the conspecific until the following day, for 24 h.

#### Unfamiliar exposure

Conspecifics were exposed during fear memory reactivation, without previous social interaction exposure.

### Contextual fear conditioning (CFC) Apparatus

#### Apparatus

The conditioning chamber consisted of an illuminated Plexiglas box, 25 × 25 cm^2^ with a metallic grid floor.

#### Training session

A contextual habituation session (5 min-exposure) was performed on all the rats twenty-four hours before conditioning training. During training, rats were placed in the chamber for 3 min and then two footshocks (0.7 mA) were administered, each lasting 2 s, with intervals of 30 s between them. They were kept in the conditioning context for an additional 30 s before being returned to their home cage.

#### Reactivation sessions

The reactivation session was performed 48 h after fear conditioning training. Memory reactivation consisted of a re-exposure of the rat (male or female) to the conditioned context for 3 min, followed by the placement or exposure of a congener (familiar or unfamiliar-male or female) in the same context for the following minutes until the reactivation session was completed - depending on the experimental protocol: 12, or 30 min (extinction session). The control groups (no social interaction: male or female) were not exposed to any social interaction and were re-exposed to the context without a conspecific.

#### Test session

24 h after the reactivation session, all groups were exposed to the conditioning chamber for 5 min (without shock or conspecifics). The percentage of freezing time during exposure was used as a measure of fear expression.

#### Test Reinstatement

Reinstatement was performed 24 h after the test session. Animals were placed in a different context, where received 2 shocks of 0.7 mA. Immediately after the footshocks, the rats were returned to their home cages. 24 h after, memory was evaluated in the conditioning chamber for 5 min.

#### Remote memory test

20 days after the last test session, animals were placed in the conditioning chamber for 5 min. The percentage of freezing time during exposure was used as a measure of return of fear memory.

### Behavioral measurement

Freezing behavior was used as a memory index, being registered with a stopwatch in real-time by an experienced observer that was unaware of the experimental conditions. Freezing was defined as the total cessation of all movements except those required for respiration (Blanchard and Blanchard, 1969) [26].

### Determination of estrous cycle

Vaginal secretion was collected from female rats by fresh vaginal lavage using a sterile pipette tip (200 μl) and 0.9% saline for subsequent determination of the estrous cycle under a light microscope. Collections were performed immediately after each experiment [27].

### Stereotaxic surgery and cannula implantation

Animals used in the experiment were anesthetized with ketamine and xylazine (75 and 10 mg/kg, respectively). The guide cannulas were implanted bilaterally at anterior/posterior (AP) 4.2 mm (from bregma), medial/lateral (ML) ± 3.0 mm, dorsal/ventral (DV) 1.8 mm, and 1.0 mm above the CA1 dorsal area. Behavior procedures were performed approximately 7 days after surgery. Animals with inaccurate cannula positions were excluded from statistical analysis.

#### Histology for cannula placement

Cannula placement was verified at the end of each experiment. After euthanasia, brains were dissected and preserved in a 4% paraformaldehyde (PFA) solution for subsequent analysis of cannula placement in the target structure.

### Statistical analysis

The data were expressed as mean ± standard error of the mean (SEM). Memory was measured by quantifying freezing behavior, expressed as a percentage of the total session time or divided into three-minute intervals (reactivation sessions). To assess normality, either the Kolmogorov-Smirnov or Shapiro-Wilk test was utilized. In one of the experiments, one group did not exhibit normal distribution; therefore, the data were transformed (log) for analysis. All experiments per group exhibited similar variance for all behaviors. For repeated measures, analysis of variance (ANOVA) was employed, followed by Tukey’s posthoc test to assess interactions between time and group. Statistical analysis was performed using GraphPad Prism 10 software. A 95% confidence level was applied to all data, and *p* < 0.05 were considered statistically significant

## Results

### Exposure of a familiar female during reactivation attenuates fear expression in males

**Fig. 1:**
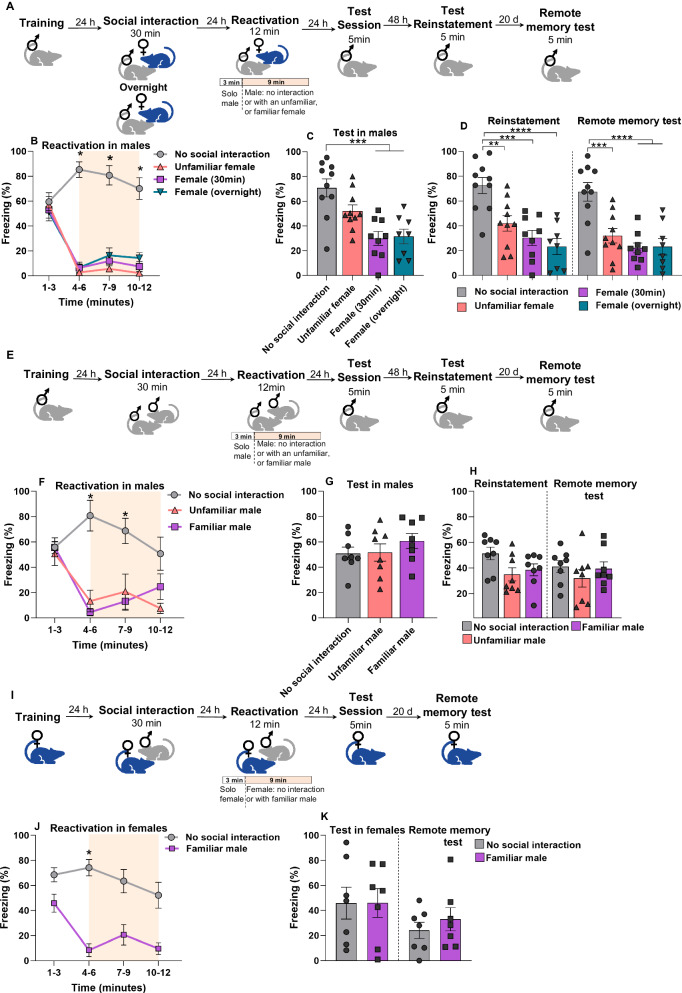
Exposure of a familiar conspecific of the opposite sex during reactivation attenuates fear memory expression in males. **A** Experimental design. Male rats were trained to CFC. 24 hours (h) after they underwent a social interaction session with a conspecific female for 30 minutes (min) or overnight. The next day fear memory was reactivated in the CFC for 12 min without female exposure (no social interaction, *n* = 10), or 3 min solo followed by 9 min with an unfamiliar female (*n* = 10), or familiar (female 30 min, *n* = 9; or female overnight *n* = 8). The test session was performed 24 h later, followed by a reinstatement test (48 h), and a remote memory test (20 days). **B** During reactivation (time in minutes), differences in freezing were observed among groups of males when females were exposed to the context. **C** In the test, differences were observed between no social interaction and males exposed to a familiar female (30 min and overnight). **D** In the reinstatement and remote memory test, males reactivated with females showed lower freezing levels compared to no social interaction (control group). **E** Experimental design. Male rats were trained to CFC. 24 h later they underwent a social interaction session with a conspecific male for 30 min. The following day, fear memory was reactivated in the CFC chamber for 12 min alone (no social interaction, *n* = 8) or 3 min solo followed by 9 min with either an a unfamiliar (*n* = 8), or the familiar male (*n* = 8). 24 h later was performed a test session, 48 h a reinstatement test, and 20 days a remote memory test. **F** During reactivation (time in minutes), differences in freezing were observed in males when the conspecific male was exposed to the context. **G** Test session, no difference was observed among the groups. **H** In the reinstatement and remote memory test, no difference was found among the groups. **I** Experimental design. Female rats were trained to CFC. 24 h later they underwent a social interaction session with a conspecific male for 30 min. The next day fear memory in females was reactivated in the CFC for 12 min without male exposure (no social interaction, *n* = 7), or 3 min solo followed for 9 min with the familiar male (*n* = 7). 24 h later was performed a test session and, 20 days later, the remote memory test. **J** During reactivation (time in minutes), differences in freezing were observed between groups of females when the familiar male was exposed to the context. **K** In the test and remote memory test, no differences were found between the groups. Bars represent mean ± SEM. *p < 0.05; **p < 0.005. ***p < 0.0005. ****p < 0.00005.

Previous research suggests that introducing rewarding stimuli during fear memory reactivation facilitates the updating of the fear memory through reconsolidation-based interventions [16]. Here, we hypothesized that the presence of an opposite-sex conspecific could serve as a stimulus capable of introducing rewarding information, thereby facilitating the attenuation of fear memory. For this, animals were fear conditioned and, 48 h later, underwent a 12-min reactivation session (3 min alone to induce memory labilization, and 9 min with the presence of a female). Animals were reactivated either alone (with no previous social interaction), with an unfamiliar female, or with a familiar female (30 min or overnight social interaction - see methods) (Fig. 1A).

We found that the presence of a female drastically reduced the freezing levels in males during reactivation compared to the control group (No Social Interaction) (Fig. 1B) [repeated-measures ANOVA, time x group interaction: *F*_(9, 63)_ = 13.4, *p* < 0.001; Tukey’s *posthoc* test revealed difference in minutes 4–6: no social interaction group vs unfamiliar female (*p* < 0.001), female (30 min) (*p* < 0.001), and female (overnight) (*p* = 0.002); minutes 7–9: no social interaction vs unfamiliar female (*p* = 0.007), female (30 min) (*p* = 0.007), and female (overnight) (*p* = 0.008); minutes 10–12: no social interaction vs unfamiliar female (*p* = 0.019), female (30 min) (*p* = 0.010), and female (overnight) (*p* = 0.029)]. These results show that the presence of a female during memory reactivation in males was able to inhibit the expression of freezing.

In the test session, only males reactivated with a familiar female (30 min and overnight) expressed lower freezing levels compared to the control group (Fig. 1C) [one-way ANOVA, F(3, 33) = 10.34, *p* < 0.0001, followed by Tukey’s posthoc revealed difference in the no social interaction group vs female (30 min) (*p* = 0.0002), female (overnight) (*p* = 0.0004), and unfamiliar female (*p* = 0.1222)]. In sum, our results in this section indicate that only the presentation of familiar females was able to attenuate fear memory response in males. It is pertinent to mention that we correlated the estrous cycle phases in familiar females during the reactivation session with the freezing test in males, and no significant differences were observed (data not shown).

To assess if the fear memory was susceptible to re-emerging after attenuation by female exposure, we conducted a session of reinstatement followed by a remote memory test (Fig. 1D). In the reinstatement protocol, the footshock was administered in a different context for a brief duration, which was not sufficient to create a memory representation (see methods). 24 h later, fear expression was tested, revealing differences between all reactivated groups with females and the control group [one-way ANOVA, F(3, 33) = 11.97, *p* < 0.0001, followed by Tukey’s posthoc revealed difference in the no social interaction group vs unfamiliar female (*p* < 0.0062), female (30 min) (*p* = 0.0002), and female (overnight) (*p* < 0.0001)]. The same pattern was observed in a remote memory test conducted 20 days later, aimed at assessing any spontaneous recovery [one-way ANOVA, F(3, 33) = 12.23, *p* < 0.0001; Tukey’s posthoc revealed difference in the no social interaction group vs unfamiliar female (*p* = 0.0006), female (30 min) (*p* < 0.0001), and female (overnight) (*p* < 0.0001)]. These results indicate that a familiar female presented during memory reactivation attenuates fear memory in a long-lasting way, suggesting that the memory content underwent an updating process. It is important to mention that these main findings were obtained after being replicated twice in our laboratory. Additionally, we implemented the identical protocol as described above, with the exception that the reactivation session extended to 15 min. Similarly, only the familiar female group exhibited a reduction in fear compared to the control group during the test, confirming the effect observed previously (data not shown).

Up to this point, we have shown that attenuation of fear memory in males is possible when a familiar conspecific of the opposite sex is presented during reactivation. We then wondered whether this effect would be possible using an animal of the same sex. Thus, we followed the same protocol described above and instead of females in the reactivation session, we used a familiar male (30 min social interaction) or unfamiliar male (Fig. 1E). In the reactivation session, males reactivated with familiar males showed lower levels of freezing compared to the control group (Fig. 1F) [repeated-measures ANOVA, time x group interaction: F_(3.51, 24.55)_ = 5.69, *p* = 0.003; Tukey’s posthoc revealed difference in the no social interaction group vs familiar males, minutes 4–6 (*p* = .0.007) and minutes 7–9 (*p* = 0.023)]. No effect was found in the test session [One way ANOVA, F_(2.21)_ = 0.8425, *p* = 0.4447] (Fig. 1G), reinstatement test [one way ANOVA, F_(2,21)_ = 3.073, *p* = 0.0675] nor the remote memory test (Fig. 1H) [one way ANOVA, F_(2,21)_ = 0.7248, *p* = 0.4961]. These results indicate that exposure with a familiar same-sex conspecific in males inhibited fear memory expression within session, but not in the subsequent tests.

So far, our results indicate that fear reactivation with an opposite-sex conspecific allows memory updating in males, with a more robust effect when a female is familiar. Next, we assessed whether this memory updating was specific to males or whether it would be also present in female. Thus, female rats were subjected to the same experimental conditions, and familiar males were placed during memory reactivation (Fig. 1I). During the reactivation session, a significant difference was found in the first minutes (Fig. 1J) [repeated-measures ANOVA, time x group interaction: F_(2.08, 12.49)_ = 5.09, *p* = 0.023; Tukey’s *posthoc* revealed difference in the no social interaction group vs familiar group, minutes 4–6 (*p* = 0.010)]. However, 24 h later, in the test session, no effect was found between the groups [Student’s *t* test, *t*_(12)_ = 0.002827, *p* = 0.9978], nor in the remote memory test (Fig. 1K) [Student’s *t* test, *t*_(12)_ = 0.7853, *p* = 0.4475]. These results indicate that the effect observed with exposure to a familiar opposite-sex conspecific was specific to male rats

### Memory destabilization and onset female exposure are necessary condition for memory updating

**Fig. 2:**
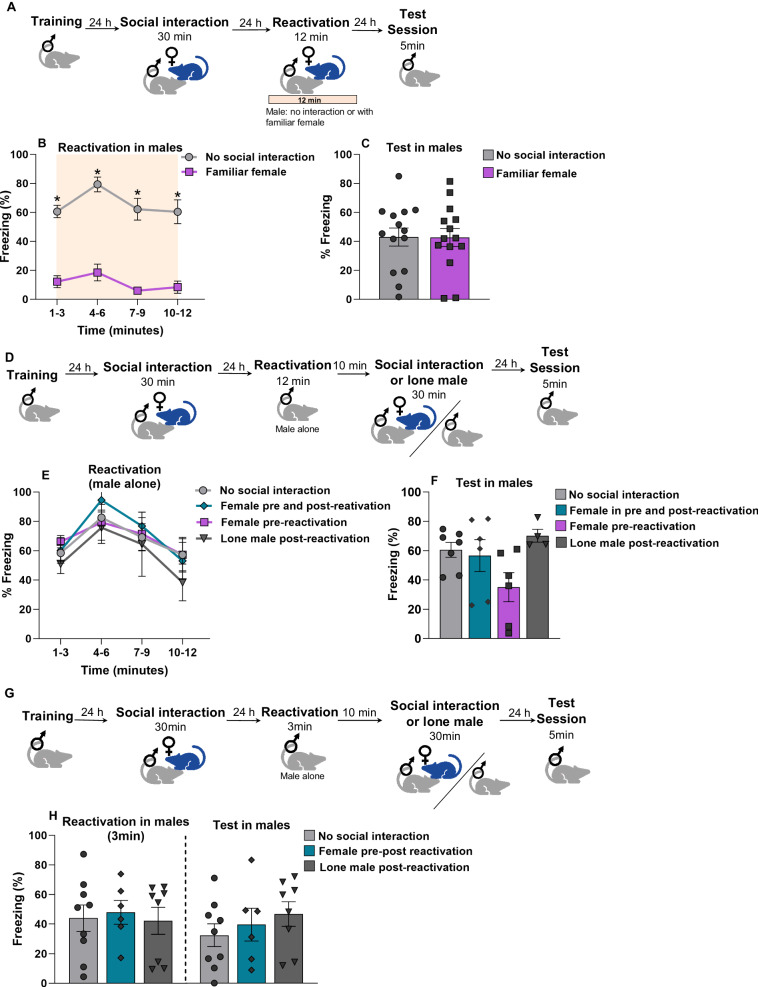
Memory destabilization and onset female exposure are necessary condition for memory updating. **A** Experimental design. Male rats were trained to CFC. 24 h later they underwent a social interaction session with a conspecific female for 30 min. The next day fear memory in males was reactivated in the CFC for 12 min without female exposure (no social interaction, *n* = 14) or with the familiar female (*n* = 14). 24 h later was performed a test session. **B** During reactivation (time in minutes), freezing levels were reduced in males exposed to females. **C** Test session, no difference was observed between the groups. **D** Experimental design. Male rats were trained to CFC. 24 h later they underwent a social interaction session with a conspecific female for 30 min. The next day fear memory in males was reactivated in the CFC for 12 min without females (no social interaction, *n* = 7). 10 min later were exposed to the context of social interaction with the familiar female (pre-post reactivation or post reactivation, *n* = 6 per group) or alone (lone male post reactivation, *n* = 4) for 30 min, control group returned to their home cages (no social interaction, *n* = 7). 24 h later was performed a test session. **E** During reactivation (time in minutes), no difference was observed among the groups. **F** Test session, no difference was observed among the groups. **G** Experimental design. Male rats were trained to CFC. 24 h later they underwent a social interaction session with a conspecific female for 30 min. The next day fear memory in males was reactivated in the CFC for 3 min (without females). 10 min later, they were exposed to the context of social interaction with the familiar female (pre-post reactivation, *n* = 6), or alone (lone male post-reactivation, *n* = 8), control group returned to their home cages (no social interaction, *n* = 9). 24 h later was performed a test session. **H** During reactivation (3 min), no difference was observed among the groups. Test session, no difference was observed among the groups. Bars represent mean ± SEM. *p < 0.05.

Memory requires to enter a labile state in order to be updated during retrieval. Thus, in our above mentioned protocols, animals were kept alone in the first 3 min of the reactivation session in order to induce memory destabilization and then, the female was inserted. We then asked whether exposing the female concomitantly with the male from the beginning of the reactivation session would also be capable to update memory to a less aversive level (Fig. 2A). During reactivation, familiar female expressed less freezing levels than the no social interaction group (Fig. 2B) [repeated-measures ANOVA, time factor: F(2.34, 30.43) = 8.477, *p* < 0.001, and group factor: F(1, 13) = 89.747, *p* < 0.001; Tukey’s posthoc revealed difference between the groups across the minutes, *p* = <0.01]. Nevertheless, 24 h later in the test session, no difference was found between the groups (Fig. 2C) Student’s *t*-test, *t*_(26)_ = 0.04057, *p* = 0.9680. This result indicates that the exposure of the conspecific from the beginning impair memory destabilization. It is possible that animals are distracted by the conspecific during retrieval, impairing the labilization and, ultimately, memory updating.

We then asked whether the exposure to a conspecific requires to be onset or would also be effective if exposed 10 min after reactivation (during the labile state induced by retrieval). To evaluate this possibility, male rats were subjected to the same training protocol. 48 h later, they were reactivated for 12 min without being exposed to females at the onset of the context. 10 min later, animals were exposed to a familiar female. Another group underwent social interaction 24 h before but not after reactivation. Finally, another group remained alone after reactivation. The control group was not subjected to any social interaction (Fig. 2D). During reactivation, all groups presented difference within session, but not among them (Fig. 2E) repeated–measures ANOVA, time factor: F_(3,9)_ = 23.46, *p* < 0.001; interaction: F_(9,27)_ = 1.43, *p* = 0.223. In the test, no difference among the groups was reported (Fig. 2F) [one-way ANOVA, F_(3,19)_ = 2.862, *p* = 0.0640]. This result evidenced that the presentation of female rats must be onset, concomitant with the reactivation of fear memory in males to promote memory updating.

Finally, we conducted a similar experiment mentioned above, but the reactivation period was 3 min instead of 12 in order to investigate whether the memory updating effect would occur in animals reactivated in the context previously associated with the footshock (thus rendering the memory labile), while the female presentation occurred in a different context 10 minutes later (Fig. 2G). No differences were found during reactivation [one-way ANOVA, F_(2,20)_ = 0.09249, *p* = 0.9120], nor in the subsequent test session [one way ANOVA, F_(2,20)_ = 0.7389, *p* = 0.4902] (Fig. 2H). Taken together, these results indicate that females need to be present at the onset and during the reactivation in order to update fear memory.

### Exposure of a familiar female impairs memory extinction

**Fig. 3:**
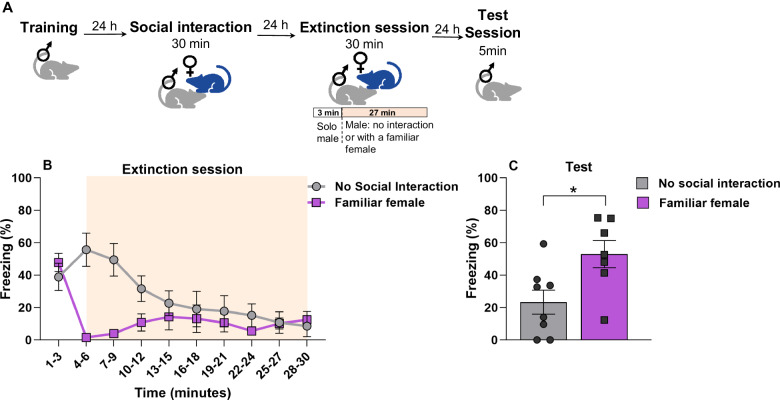
Exposure of a familiar female impairs extinction memory. **A** Experimental design. Male rats were trained to CFC. 24 h later they underwent a social interaction session with a conspecific female for 30 min. The following day, the males were taken to an extinction session in the CFC for 30 min, alone (no social interaction, *n* = 8) or 3 min solo followed by the exposure of the familiar female for 27 min (*n* = 7). **B** During the extinction session (time in minutes). **C** Test session, no difference was observed between the groups. Bars represent mean ± SEM. *p < 0.05.

Next, we wondered whether a familiar female exposure during an extinction session would affect extinction learning. Thus, a set of male rats underwent the same training and social interaction protocol. During the long reactivation session (extinction training) the familiar female was placed at minute 3 and remained for 27 min until the extinction session was completed (Fig. 3A). During extinction training, both groups presented difference within session (Fig. 3B) [repeated-measures ANOVA, time x group interaction: F_(9,54)_ = 9.64, *p* < 0.001]. In the session test, the familiar female group presented a higher freezing level (Fig. 3C) [student’s *t* test, *t*_(13)_ = 2.660, *p* = 0.0196]. Thus, the males that underwent extinction training with familiar females showed extinction impairment (Fig. 3C).

### Hippocampal D1/D5 receptors and oxytocin are involved in fear memory updating

Dopamine release is a fundamental element of the brain’s reward circuitry, with major projections originating from the Ventral Tegmental Area (VTA) to the Nucleus Accumbens (NA). However, it’s important to note that the VTA also sends connections to various other regions, including the prefrontal cortex, amygdala, and hippocampus [28–30]. The hippocampus plays a crucial role as a mediator in reinforcing memory encoding based on the valence of stimuli and in registering novel experiences [31, 32]. Dopamine receptors (D1/D5) in the hippocampus are involved in the attenuation of fear memory in males with female exposition [20], appetitive behavior, and sexual motivation [33, 34]. Here, we evaluated whether blocking the D1/D5 receptors in the hippocampus prevents this rewarding state in males with females and, consequently, memory updating. For this purpose, a batch of males underwent surgery to implant cannulas into the hippocampus. One week later they were subjected to the same behavior protocol described above. Animals received bilateral intra-CA1 infusions of the D1/D5 receptor antagonist SCH23390 (SCH;1 µg/side) before reactivation. In the reactivation session, no differences were found between groups [repeated measures ANOVA, time x group interaction: F_(1.50,8.97)_ = 0.648, *p* = 0.503, group: F_(1,6)_ = 1.316, *p* = 0.295, time: F_(1.32,7.90)_ = 3.995, *p* = 0.074]. SCH group presented higher freezing levels in the test [Student’s *t* test, *t*_(14)_ = 2.921, *p* = 0.0119]. Taken together, these results indicate that inactivating D1/D5 receptors in the hippocampus during retrieval memory prevented the effect of memory updating in the reactivated group with female rats. Another mechanism that could be implicated in social interaction is the release of oxytocin (OXT). Indeed, hippocampal OXT receptors play a pivotal role to social recognition [35], and participates in rewarding social interactions [36–38]. Here we used an oxytocin receptor antagonist in the hippocampus during the reactivation session to assess whether its blockade would impair fear memory attenuation in males. To conduct this experiment, males went through the same protocol described previously, and Atosiban or vehicle was administered before reactivation. No differences were registered between groups in the reactivation session [repeated-measures ANOVA, time x group: F_(1.48,5.93)_ = 0.256, *p* = 0.720, group: F_(1,4)_ = 6.886, *p* = 0.059, time: F_(2.30,9.21)_ = 3.708, *p* = 0.062]. Nonetheless, atosiban group presented higher freezing levels in the test [Student’s *t* test, *t*_(9)_ = 4.180, *p* = 0.0024].

These results show that blocking oxytocin receptors in the hippocampus during reactivation impaired the fear memory attenuation effect. Then, we decided to verify whether the increase of the oxytocin levels in the reactivation is sufficient to reduce freezing levels in the test. Thus, intrahippocampal oxytocin was infused prior to reactivation to simulate the interaction effect with the female (females were not exposed in this experiment). No significant effects were found between groups during the reactivation session [repeated-measures ANOVA, time x group interaction: F_(1.50,7.49)_ = 0.6176, *p* = 0.520, group: F_(1,5)_ = 0.0916, *p* = 0.774, time: F_(1.94,9.70)_ = 1.2378, *p* = 0.331]. However, OXT group expressed lower freezing levels in the test [Student’s *t* test, *t*_(11)_ = 2.668, *p* = 0.0219]. This result indicates that OXT in the hippocampus is associated with a reduction in fear memory through female exposure during memory reactivation in males.

## Discussion

**Fig. 4:**
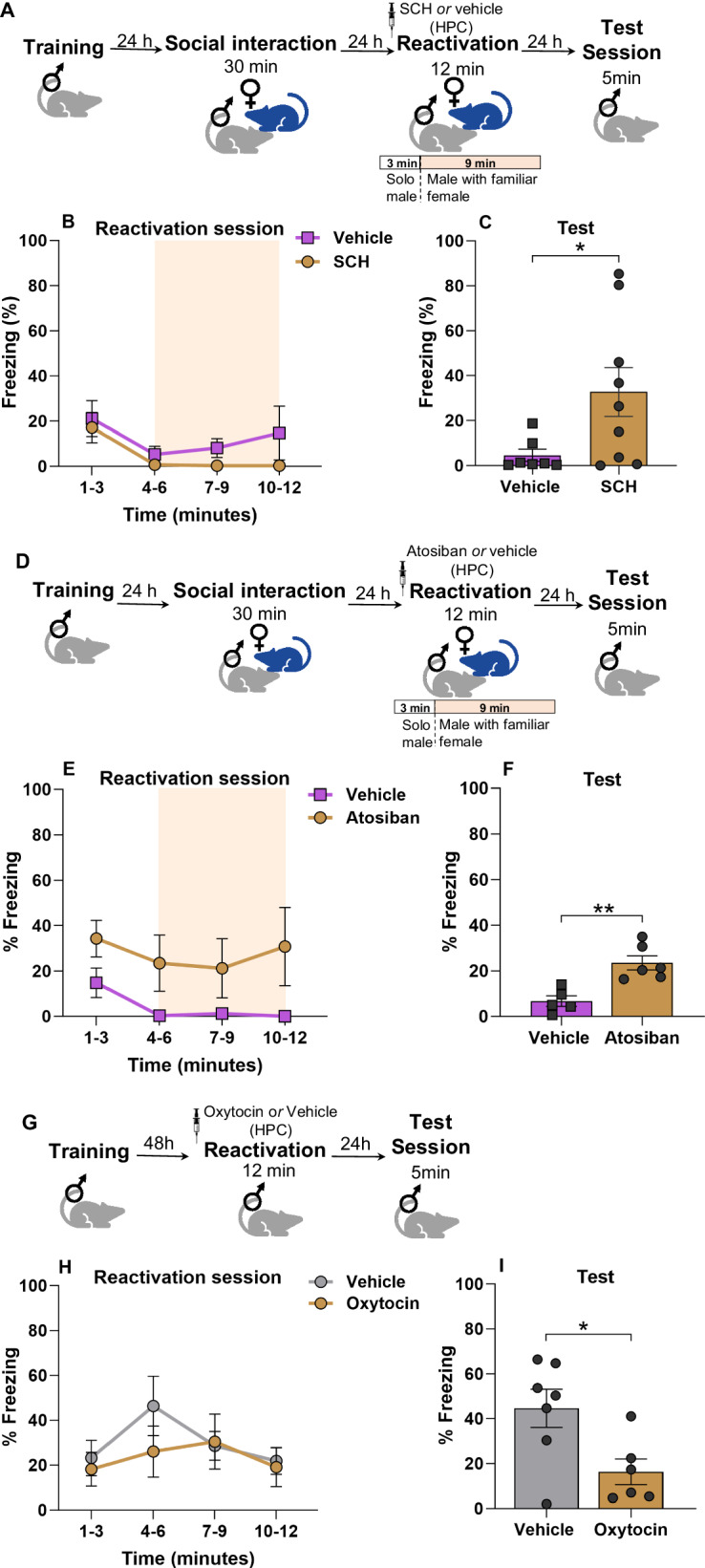
Hippocampal D1/D5 receptors and oxytocin are involved in the updating of fear memory with exposure to a familiar opposite-sex conspecific in males. **A** Experimental design. Male rats were trained to CFC. 24 h later they underwent a social interaction session with a conspecific female for 30 min. The following day, fear memory in males was reactivated in the CFC for 12 min (3 min solo, 9 min with the familiar female). SCH (*n* = 9) or its vehicle (*n* = 7) was infused into HPC 20 min before the reactivation session. 24 h later was performed a test session. **B** During reactivation (time in minutes), no difference was observed between the groups. **C** Test session, the SCH group expressed a higher freezing level compared to vehicle. **D** Experimental design. Male rats were trained to CFC. 24 h later they underwent a social interaction session with a conspecific female for 30 min. The following day, fear memory in males was reactivated in the CFC for 12 min (3 min solo, 9 min with the familiar female). Atosiban (*n* = 6) or its vehicle (*n* = 5) was infused into HPC 20 min before the reactivation session. 24 h later was performed a test session. **E** During reactivation (time in minutes), no difference was observed between the groups. **F** Test session, the Atosiban group expressed a higher freezing level compared to the control. **G** Experimental design. Male rats were trained to CFC. 48 h later fear memory was reactivated in the CFC for 12 min. Oxytocin (*n* = 6) or its vehicle (*n* = 7) was infused into HPC 25 min before the reactivation session. 24 h later was performed a test session. **H** During reactivation (time in minutes), no difference was observed between the groups. **I** Test session, the Oxytocin group expressed a higher freezing level compared to the control. Bars represent mean ± SEM. *p < 0.05; **p < 0.005.

In this study, we evaluated the effect of a natural rewarding stimulus (social interaction) during fear memory reactivation in rats. We found that presentation of a familiar female during fear memory reactivation (12 min) reduced freezing expression in the session test, without presenting reinstatement or a remote memory effect (Fig. 1). We also showed that it is required to be 3 min alone in the exposure context before presenting the female, and this has to be onset the conditioned context (Fig. 2). Furthermore, we demonstrated that this social interaction between males and females during extinction training impaired acquisition of extinction memory (Fig. 3). Finally, our results indicate that dopamine and oxytocin receptors in hippocampus mediate the memory updating effect that leads to fear attenuation (Fig. 4).

The reactivation of fear memories in conjunction with the presentation of rewarding stimuli has been linked to a decrease in fear expression in subsequent tests. This reduction occurs during the labile state induced after reactivation, where memories are open to modification, allowing for the integration of positive valence information to update their content [3, 16, 39]. In this study, our hypothesis was that exposure to a natural reward stimulus, such as social interaction with a conspecific, during the plastic state induced by reactivation would lead to a reduction in fear expression.

We have observed in all experiments (regardless of sex or familiarity) that the presence of a conspecific reduces the expression of freezing within reactivation session (Figs. 1–3). This fear reduction has been described because of the social buffering effect [40–42]. However, it is essential to acknowledge that animals may also experience a decrease in fear due to distraction when introduced to a companion rat, leading to a reduction in freezing response. We found that attenuation of fear memory in males was more consistent when a familiar female was used. Accordingly, it has been shown that presenting a familiar conspecific is more effective than unfamiliar to reduce freezing expression [23]. As depicted in Fig. 1D, the familiar female promotes a fear reduction across all tests, while the introduction of an unfamiliar female shows a fear attenuation in some of them, suggesting a mild updating effect. This phenomenon could be attributed to the heightened effectiveness of familiar conspecific presence in alleviating stress responses and providing social buffering during reactivation [23].

Our results are in accordance with other studies using a rewarding/positive-valence stimuli during reactivation in order to reduce fear memory. For instance, fruit-loops cereal [16] caffeine [17], or methylphenidate [15] presented during fear memory reactivation leads to the attenuation of fear expression by incorporating this new information in the background of the original aversive memory. It is important to mention that our results seem to be permanent since any effect was found in the remote memory or reinstatement tests. Our results could also be interpreted as a counterconditioning intervention, as a novel stimulus with a positive valence was introduced during memory retrieval, thereby updating the memory valence to a less aversive state [43]. Two alternative explanations could potentially account for our current findings: Firstly, an interference effect triggered by the female during the labile state induced by retrieval may disrupt reconsolidation. Secondly, we cannot dismiss the possibility that the presence of the female is fostering a new, positive association with the context. Consequently, during the test phase, animals may recall both experiences, supported by distinct engrams. As a result, the negative fear-context association may be diluted by the positive female-context association, leading to a less aversive representation of the context and then, reducing freezing behavior.

It is important to note that when animals were exposed together right from the start of the reactivation process, no reduction in fear was observed (Fig. 2C). It’s conceivable that the presence of the female from the beginning hinder retrieval, a process essential for destabilization. This result suggests that this interaction needs to occur after approximately 3 min, which is the time required to induce the destabilization of the memory trace and make it susceptible to reconsolidation (De Olivera Alvares et al., 2013; [3, 11, 39]). A similar pattern has been observed in the reactivation-extinction procedure, wherein the permanent updating process—resistant to spontaneous recovery, renewal, and reinstatement—occurs only when a brief reactivation is presented shortly before extinction [24]. Our results could also be interpreted as a counterconditioning intervention, as a novel stimulus with a positive valence was introduced during memory retrieval, thereby updating the memory valence to a less aversive state [43].

Furthermore, it is crucial for this interaction to be contextually contingent. When animals were exposed to the context first and then introduced to the conspecific in another place, no memory updating was observed (Fig. 2F). It is also worth noting the absence of any effect on fear reduction in the test when a male conspecific was presented during the reactivation session. Similarly, when a female was fear-conditioned and a male was introduced during reactivation, no fear reduction was shown. In both scenarios, some reduction in fear was observed during the reactivation session itself, but this reduction was not maintained in the subsequent tests. This outcome suggests that the animals might have merely been distracted by the presence of another rat, and no substantial emotional remodeling occurred under these conditions. The reason why males respond differently in the presence of females (as opposed to other combinations) remains unknown. It is possible that when a male is introduced to another male or when a male is presented to a female, a stressful rather than rewarding dynamic occurs. Another possibility is that females find same-sex interactions less rewarding than males [44]. Future studies in females are needed to explore the implications of this phenomenon in same-sex interactions.

Social interaction has universally been characterized as a rewarding experience across species, transcending sex and age. These favorable outcomes are intricately connected to the functioning of dopamine mechanisms and the release of oxytocin, which significantly reinforce the behavioral motivation for engaging with conspecifics and the subsequent encoding of these interactions [45–47]. These effects have been extensively explored and documented in various behavioral tasks using animal models [48]. In this study, we demonstrated that blocking the oxytocin receptors in the hippocampus before reactivation in the presence of a female impairs the reduction of fear memory. Also, replicating this effect by exogenously infusing oxytocin in the HPC during a fear memory reactivation was able to reduce fear memory without the presence of a female. These findings strongly suggest that oxytocin is both necessary and sufficient to facilitate memory updating through social interactions during retrieval. Indeed, previous research has linked oxytocin to the modulation of fear through social memory (Guzmán et al., 2014) [49] through its rewarding properties [50]. However, we cannot rule out the possibility that the oxytocin effect depicted in Fig. 4I–K might be unrelated to the updating effect.

We also observed that the infusion of the D1/D5 receptor antagonist (SCH) into the hippocampus before reactivation with social interaction effectively blocked the memory-updating effect. Interestingly, our findings align with previous research, which demonstrated that exposure of females to males immediately after fear conditioning leads to a reduction in freezing response [20]. This effect can be prevented by the D1/D5 receptor antagonist within the hippocampus [20]. Crucially, it is essential to note that our findings should not be interpreted as consequence of direct sexual interaction, since no spermatozoa were found in the vaginal lavage of the females when collected after reactivation.

We also examined the impact of female exposure during extinction training. Interestingly, we observed an impairment in the extinction process. It is our belief that the presence of a conspecific might serve as a distracting factor that hinders the acquisition of extinction.

In summary, our findings offer compelling evidence supporting the significant impact of social interaction and gender-related distinctions between conspecifics on the attenuation and updating of fear memories. Moreover, our results highlight the crucial roles played by oxytocin and dopaminergic receptors in the hippocampus. This discovery paves the way for exploring novel avenues that could potentially integrate non- pharmacological interventions into the treatment of traumatic memories and anxiety disorders such as phobias or PTSD.

### Supplementary information

Supplemental material

## Data Availability

The data that support the findings of this study are available from the corresponding author upon request.
