# Supplementary material for: Social interaction-induced fear memory reduction: exploring the influence of dopamine and oxytocin receptors on memory updating
Source: Transl Psychiatry. 2024 Jun 6;14:242. doi: 10.1038/s41398-024-02955-3 (PMC11156639; doi:10.1038/s41398-024-02955-3)

(A) Illustration of the target area displaying representative images depicting the cannula location of each animal and groups. Drawings in the coronal plane anterior (A) posterior (P) ranging from -3.25 to 3.70 mm were adapted from the Brain Maps Atlas by Swanson (2004). **(B)** Representative image of cannula placement in the hippocampus: photomicrograph depict cannula locations at 4X magnification, stained using the Nissl technique.

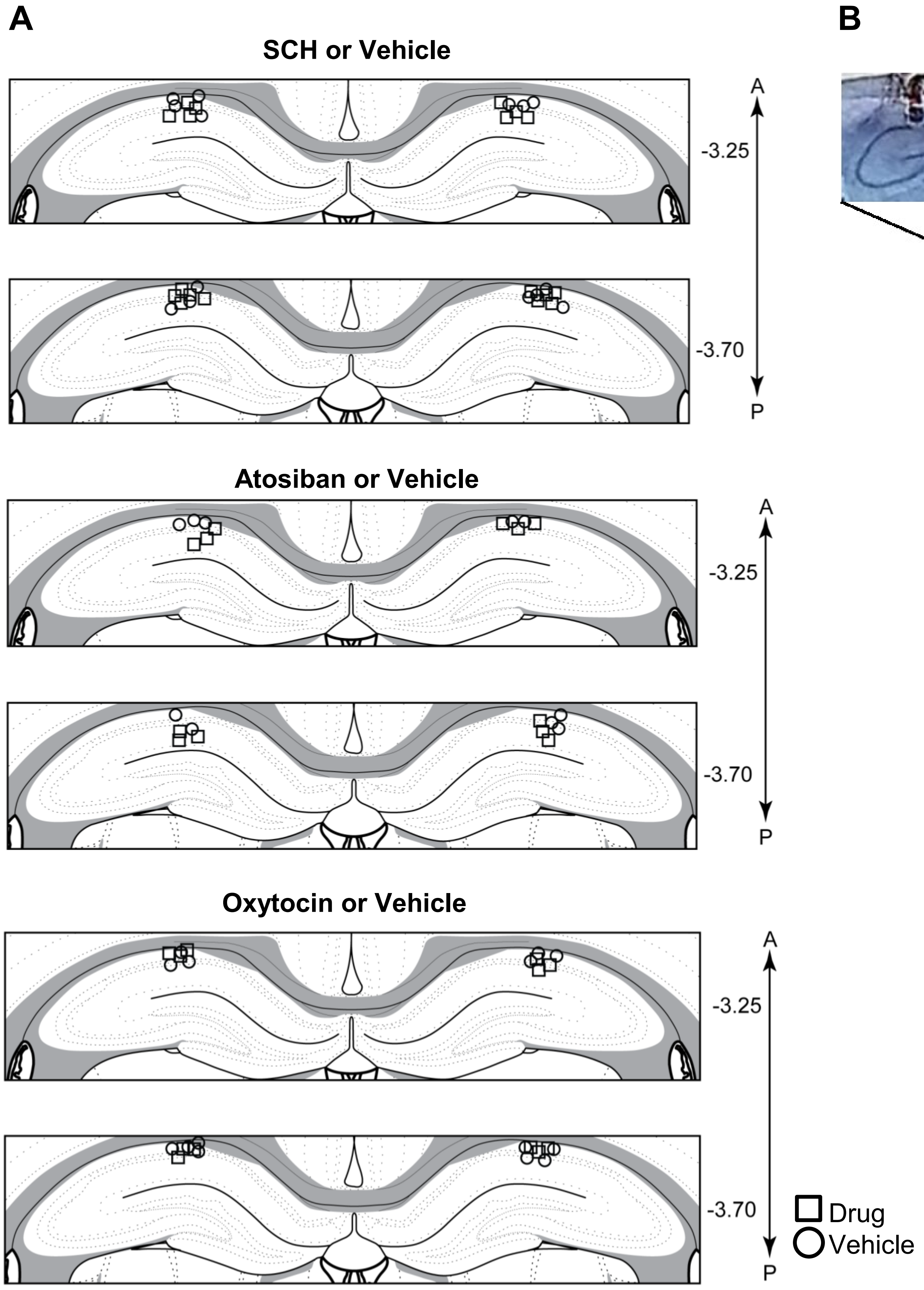

Supplement: Supplementary file 1 — Supplemental material [file 41398_2024_2955_MOESM1_ESM.pdf]
